# VLPs Displaying a Single L2 Epitope Induce Broadly Cross-Neutralizing Antibodies against Human Papillomavirus

**DOI:** 10.1371/journal.pone.0049751

**Published:** 2012-11-19

**Authors:** Ebenezer Tumban, Julianne Peabody, Mitchell Tyler, David S. Peabody, Bryce Chackerian

**Affiliations:** Department of Molecular Genetics and Microbiology, University of New Mexico School of Medicine, Albuquerque, New Mexico, United States of America; The University of Hong Kong, Hong Kong

## Abstract

**Background:**

Virus-like Particles (VLPs) display can be used to increase the immunogenicity of heterologous antigens. Here, we report the use of a bacteriophage MS2-based VLP display platform to develop a monovalent vaccine targeting a broadly neutralizing epitope in the minor capsid protein human papillomavirus (HPV) that provides broad protection from diverse HPV types in a mouse pseudovirus infection model.

**Methodology/Principal Findings:**

Peptides spanning a previously described cross-neutralizing epitope from HPV type 16 were genetically inserted at the N-terminus of MS2 bacteriophage coat protein. Three of the four recombinant L2-coat proteins assembled into VLPs. L2-VLPs elicited high-titer anti-L2 antibodies in mice, similar to recombinant VLPs that we had previously made in which the L2 peptide was displayed on a surface-exposed loop on VLPs of a related bacteriophage, PP7. Somewhat surprisingly, L2-MS2 VLPs elicited antibodies that were much more broadly cross-reactive with L2 peptides from diverse HPV isolates than L2-PP7 VLPs. Similarly, mice immunized with L2-MS2 VLPs were protected from genital and cutaneous infection by highly diverse HPV pseudovirus types.

**Conclusion/Significance:**

We show that peptides can be displayed in a highly immunogenic fashion at the N-terminus of MS2 coat protein VLPs. A VLP-based vaccine targeting HPV L2 elicits broadly cross-reactive and cross-protective antibodies to heterologous HPV types. L2-VLPs could serve as the basis of a broadly protective second generation HPV vaccine.

## Introduction

It is estimated that nearly 500,000 individuals worldwide are affected with Human Papillomavirus (HPV)-associated cancer every year [1]. There are over 120 different HPV genotypes that have been identified. The most common HPV-associated cancer, cervical cancer, is associated with infection by one of a subset of 15–18 carcinogenic “high-risk” HPV types. Two of the high-risk types, HPV16 and 18, account for approximately 70% of cases of cervical cancer [2], [3]. HPV16 infection is also a significant and growing cause of oropharyngeal cancer.

The current HPV vaccines (Gardasil® and Cervarix®) are derived from virus-like particles (VLPs) consisting of the HPV L1 major capsid protein. Gardasil® contains L1-VLPs derived from HPV16 and 18, as well as VLPs derived from two low-risk HPVs associated with genital warts (HPV6 and 11). Cervarix® only contains HPV16 and HPV18 L1-VLPs. Both vaccines induce high-titer protective antibodies to the HPV types included in the vaccine, but unfortunately these antibodies are type-specific, meaning that vaccination provides very little cross-protection to other “high-risk” HPV types [4], [5], [6], [7]. Given that these vaccines offer about 70% protection from cervical cancer [8], [9], there is still a need for both cervical Pap screening and/or HPV DNA testing in order to diagnose high-risk infections with those carcinogenic HPV types not covered by the vaccines.

Second-generation HPV vaccines providing broader protection against the spectrum of carcinogenic “high-risk” HPV types might permit further reductions in screening intensity in developed nations. Merck has indicated that a nonavalent L1-VLP vaccine is currently in clinical trials [10]. However, given that the current HPV vaccines are amongst the most expensive on the market, it remains to be seen whether the nonavalent vaccine will be applicable in resource-poor settings. Second generation vaccines with lower cost and other features (without a cold-chain requirement and needing fewer doses) would be especially useful for the developing world.

As an alternative tactic to broaden the efficacy of HPV vaccines, a number of groups have exploited the presence of highly-conserved cross-neutralizing epitopes in the L2 minor capsid protein of HPV to develop second-generation L2-based pan-HPV vaccines [11], [12], [13], [14], [15], [16], [17]. Because L2 neutralizing epitopes are thought to be exposed only transiently during HPV infection, natural infection fails to induce anti-L2 antibody responses. Thus, there appears to be little selective pressure for antigenic variation in L2. Indeed, numerous studies have shown that peptides representing portions of the N-terminus of L2 induce antibodies that are capable of neutralizing infection by diverse HPV types [16], [17], [18], [19]. Although recombinant L2 protein is poorly immunogenic and induces low titer neutralizing antibodies, its immunogenicity can be increased by various formulations. For example, we have recently shown that the multivalent display of an L2 epitope on the surface of a recombinant bacteriophage PP7 coat protein VLP can dramatically increase the immunogenicity of the L2 peptide [12].

Although the sequence of the N-terminal domain of HPV L2 is relatively conserved amongst HPV types, there is some heterogeneity that can limit the breadth of reactivity of antibodies elicited against individual HPV L2 sequences. For example, a concatenated multimeric L2 fusion protein, which contains amino-terminal L2 peptides derived from 3 to 22 HPV types elicits broader neutralizing antibody responses than similar immunogens that contain L2 sequence from a single HPV type [13]. Similarly, we showed that mice immunized with PP7 VLPs displaying a peptide from HPV16 L2 elicited antibodies that bound strongly to HPV16 and HPV18 L2 peptides, but poorly to L2 peptides from other HPV types (HPV1, 5, & 6). To broaden the protection conferred by vaccination, we immunized mice with a mixture of eight L2-PP7 VLPs displaying L2 sequence from diverse HPV types. This mixture of eight L2-PP7 VLPs elicited a broader anti-L2 immune response in mice as well as broad protection from genital challenge with HPV pseudovirions (PsV) [12]. While this demonstrated the many advantages to the bacteriophage VLP strategy – low production costs, high stability, and robust immune response to low doses of antigen – the requirement for a multicomponent vaccine lessens the value of targeting a putatively broadly neutralizing epitope in L2.

In our previous study L2 peptides were inserted into a constrained ß-hairpin structure (the AB-loop) on the surface of the PP7 coat protein. Because of these structural constraints, we were somewhat concerned that this context may not have been ideal for display of L2 peptides. Therefore, we explored a different display site at the N-terminus of the coat protein of an alternate VLP display platform based on the bacteriophage MS2. Somewhat surprisingly, immunization with MS2 VLPs displaying an HPV16 L2 peptide at the N-terminus of coat protein elicited much broader cross-reactive antibodies compared to the same epitope displayed on the AB-loop of PP7 VLPs. Moreover, mice immunized with MS2 VLPs displaying a HPV16 L2 peptide were strongly protected against vaginal and intradermal challenge with HPV pseudovirions representing diverse types. These data demonstrate that a single recombinant VLP displaying an L2 peptide can provide broad protection against HPV infection.

## Results

### Display of L2 Peptides at the N-terminus of MS2 Coat Protein

**Figure 1:**
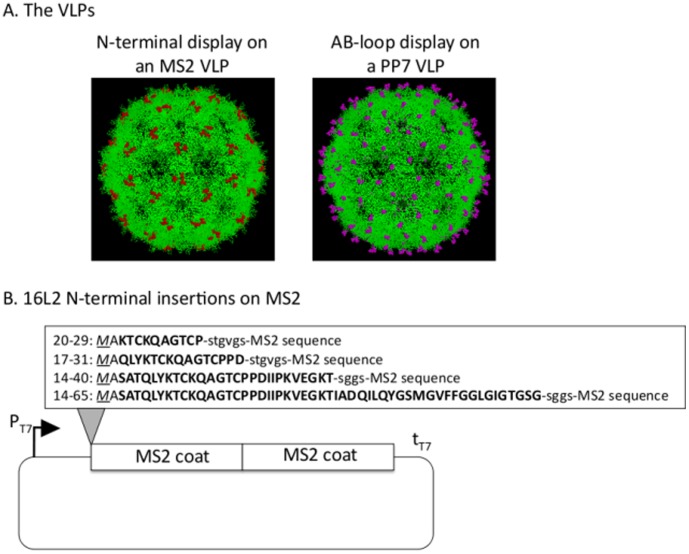
Peptide display sites on MS2 and PP7 VLPs. (A) RasMol-generated structures of MS2 VLPs (left panel) and PP7 VLPs (right panel). Red and purple colors depict the locations of the N-terminus of MS2 VLPs and the AB-loop of PP7 VLPs, respectively. In our recombinant VLPs, only 90 of the 180 coat proteins/VLP display the L2 peptide. B) Amino acid sequences of 16L2 peptides (capital bold characters; amino acids 20–29, 17–31, 14–40, and 14–65) inserted at the N-terminus of the single-chain dimer of MS2 coat protein. The start codon in capital italicized characters is underlined. Linker sequences are shown in small characters.

The family of single-stranded RNA bacteriophage is a versatile platform for displaying heterologous epitopes. We have previously shown that insertions of diverse short peptides into a surface-exposed constrained loop (the AB-loop) on the coat protein of both bacteriophage MS2 and PP7 are compatible with VLP assembly [12], [20], [21]. Recombinant VLPs display heterologous peptides in a highly multivalent format and, upon immunization, can elicit high-titer antibody responses against the inserted peptide. We were interested in assessing whether peptides could also be displayed at the N-terminus of bacteriophage MS2 coat protein. As shown in Figure 1A, the N-terminus of MS2 is displayed prominently on the surface of MS2 VLPs, suggesting that it may be a useful site for immunogenic display. The N-termini of RNA bacteriophage coat proteins are close together at the three-fold axis of their icosahedral shell, which may present difficulties in inserting peptides at this site. Encouragingly, however, previous work has shown that N-terminal insertions to the coat protein of AP205, a related RNA bacteriophage, are compatible with VLP assembly and antigenic display [22].

**Figure 2:**
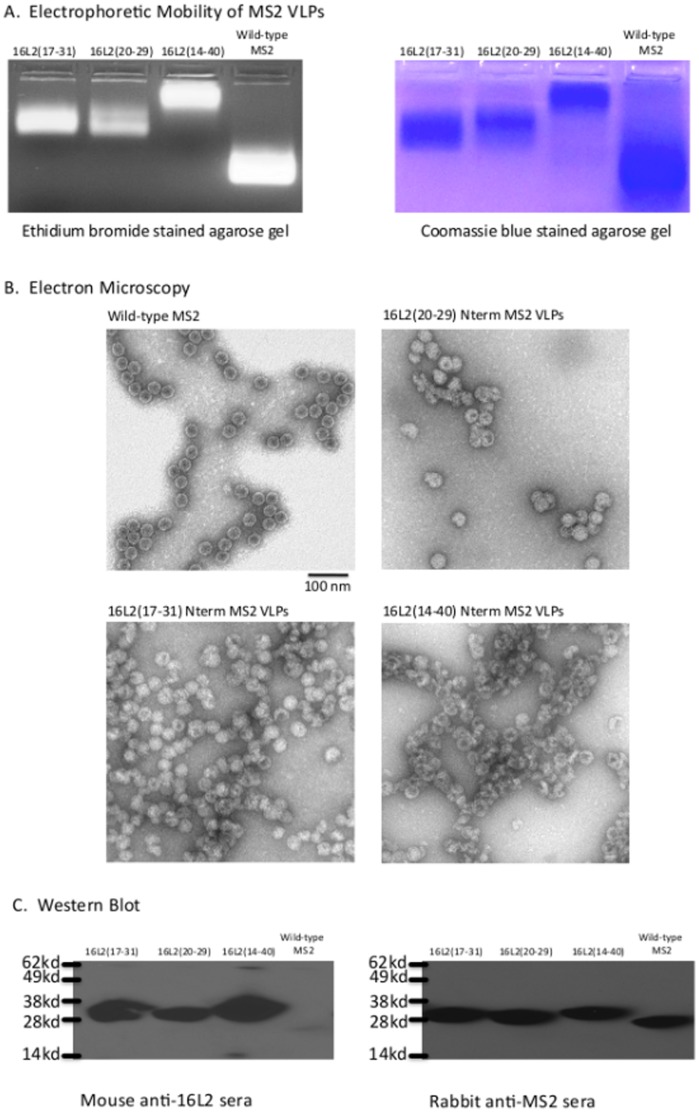
Characterization of recombinant 16L2-MS2 coat proteins. (A) An ethidium bromide stained-agarose gel showing encapsidated RNAs (left panel) and the same gel (right panel) stained with coomassie blue, showing the coat proteins at the same position of encapsidated RNAs. (B) Representative transmission electron micrographs of purified wild-type MS2 VLPs, 16L2(20–29) Nterm MS2 VLPs, 16L2(17–31) Nterm MS2 VLPs, and 16L2(14–40) Nterm MS2 VLPs. Images are magnified 70,000x. (C) Western blot analysis of recombinant L2-VLPs. Blots were probed with either mouse anti-16L2 sera (left panel) or rabbit anti-MS2 sera (right panel).

We took advantage of an engineered version of the MS2 coat protein to make N-terminal recombinants. Genetic fusion of two copies of the MS2 coat protein forms a single-chain dimer version of the protein that, when expressed, spontaneously self-assembles into VLPs that are more stable than VLPs formed from coat protein monomers [23]. Sequences representing amino acids 20–29, 17–31, 14–40, and 14–65 of HPV16 L2 were genetically inserted at the N-terminus of the single-chain dimer of MS2 coat protein (Figure 1B). Out of the four recombinant 16L2 Nterm MS2 coat proteins that were expressed, three of them [L2(20–29), L2(17–31), and L2(14–40)] assembled into VLPs that are predicted to display 90 copies of the L2 peptide per VLP. Coat protein displaying L2 amino acids 14–65 failed to form VLPs (data not shown). VLP assembly was confirmed by electrophoresis on an agarose gel as well as by transmission electron microscopy (TEM). Figure 2A shows an agarose gel in which purified VLPs were subjected to electrophoresis and then stained with ethidium bromide or coomassie blue. In this assay VLPs migrate through the gel by virtue of their surface charge with different peptide inserts conferring differences in mobility. The co-migration of encapsidated RNA (stained with ethidium bromide) and protein (stained with coomassie blue) strongly indicates VLP assembly. The morphology of these L2-MS2 VLPs was confirmed by TEM (Figure 2B). All of the VLPs were similar in diameter to wild-type MS2 VLPs. It is our impression that the MS2 VLPs displaying the L2 peptides were somewhat less regular in their morphology. All three L2-MS2 VLPs contain the L2 peptides on the VLPs as shown by Western blot (Figure 2C). The L2-MS2 coat proteins reacted with mouse anti-L2 polyclonal sera as well as with rabbit anti-MS2 sera.

### MS2 VLPs Displaying L2 Peptides are Highly Immunogenic

**Figure 3:**
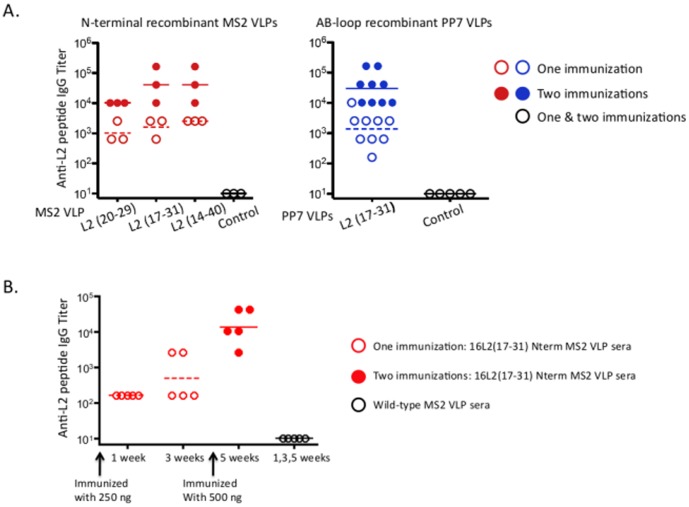
Immunogenicity of L2-MS2 and L2-PP7 VLPs in mice. (A) Groups of Balb/c mice were immunized twice intramuscularly at two-weeks interval with 5 µg each of the three 16L2-MS2 VLPs (displaying epitopes 20–29, 17–31, and 14–40) or control MS2 or with 16L2(17–31) AB-loop PP7 VLPs or control PP7 VLPs with IFA. Sera were collected after two weeks following each immunization. (B) Balb/c mice were immunized i.m. with 250 ng of 16L2(17–31) Nterm MS2 VLPs or control MS2 VLPs (without IFA) and four weeks later, both groups of mice were boosted with 500 ng of their respective VLPs without IFA. Sera was collected at weeks one, three, and five. In both cases, IgG titers were determined by end-point titration ELISA using 16L2 peptide (amino acid 14–40) as target antigen. Open red and blue circles represent titers after one immunization; broken red and blue lines represent geometric mean IgG titers after one immunization. Red- & Blue-filled circles represent titers after two immunizations and the solid lines represent geometric mean IgG titers after two immunizations. White-filled black circles represent both one and two immunizations.

To test the immunogenicity of the 16L2 Nterm MS2 VLPs, groups of mice were immunized twice with 5 µg of each of the three L2-MS2 VLP constructs. As controls, we also immunized groups of mice with recombinant PP7 VLPs which displayed the same epitope in the AB-loop of coat protein (previously described, [12]) or wild-type VLPs. Antibody responses to HPV were assessed by ELISA using conformationally constrained, disulfide-bond linked peptides representing L2 amino acids 14–40 as the target antigen. All of the L2-displaying VLPs induced high-titer anti-L2 antibody responses. End-point dilution antibody titers averaged 10^3^ after a single immunization and>10^4^ after boosting (Figure 3A). L2-MS2 VLPs were immunogenic at very low doses. Mice had detectable (∼10^2^) anti-L2 antibody titers after a single 250 ng dose of VLPs. Boosting with 500 ng of VLPs increased these titers to ∼10^4^ (Figure 3B).

**Figure 4:**
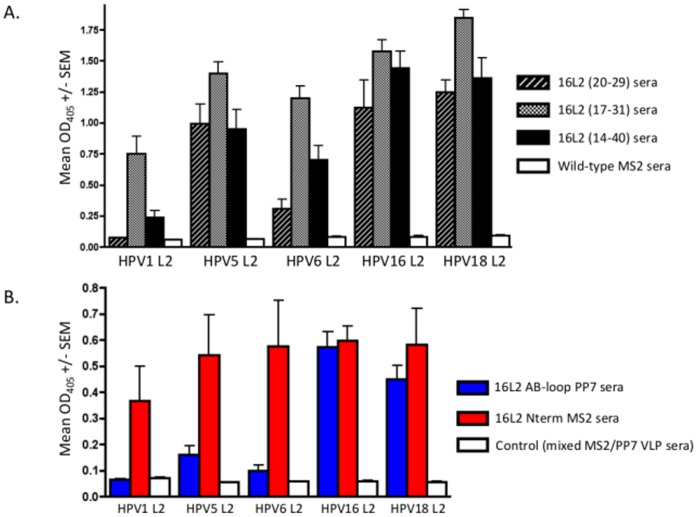
Analysis of cross-reactivity of antiserum to L2 peptides from diverse HPV types. (A) Cross-reactivity of 16L2 Nterm MS2 VLP sera with diverse HPV L2 peptides. ELISA plates were coated with 500 ng of streptavidin-conjugated L2 peptides (amino acid 14–40) from HPV 1, 5, 6, 16, and 18. Diluted sera (1∶640) from mice immunized with 16L2(20–29) Nterm MS2 VLPs or 16L2(17–31) Nterm MS2 VLPs or 16L2(14–40) Nterm MS2 VLPs was reacted with the peptides in ELISA plates. Shown are the average optical densities (ODs) at 405 nm for 3 mice. Error bars represent standard error of the mean (SEM). (B) Comparison of cross-reactivity levels of 16L2(17–31) Nterm MS2 VLP sera and 16L2(17–31) AB-loop PP7 VLP sera with diverse HPV L2 peptides. ELISA plates were coated with peptides as described above and then reacted with sera from mice immunized with 16L2(17–31) Nterm MS2 VLPs or 16L2(17–31) AB-loop PP7 VLPs or MS2/PP7 VLPs at a 1∶2,560 dilution. Average ODs (405 nm) from 3 mice are shown. Error bars represent SEM.

We measured the ability of antibody from these mice to cross-react with synthetic disulfide-constrained L2 peptides representing amino acids 14–40 from diverse HPV types (HPV 1, 5, 6, 16, & 18). As shown in Figure 4A, IgG from mice immunized with 16L2(17–31) Nterm MS2 VLPs showed the broadest cross-reactivity to the five L2 peptides tested. Sera from mice immunized with 16L2(17–31) Nterm MS2 VLPs were chosen for side-by-side cross-reactivity comparison with 16L2(17–31) AB-loop PP7 VLPs. As shown in Figure 4B, the display of epitope 17–31 on the N-terminus of MS2 bacteriophage induces more broadly cross-reactive antibodies to diverse L2 peptides than the display on the AB-loop of PP7. Notably, mice immunized with 16L2(17–31) Nterm MS2 VLPs elicited antibodies that bound to the HPV1 L2 peptide, which has the most divergent sequence among the L2 peptides tested. We also tested, by ELISA, whether anti-L2 sera could bind to HPV16 PsV. End-point dilution titers using PsV correlated with the anti-L2 peptide titers but were about 4-fold lower (data not shown).

**Figure 5:**
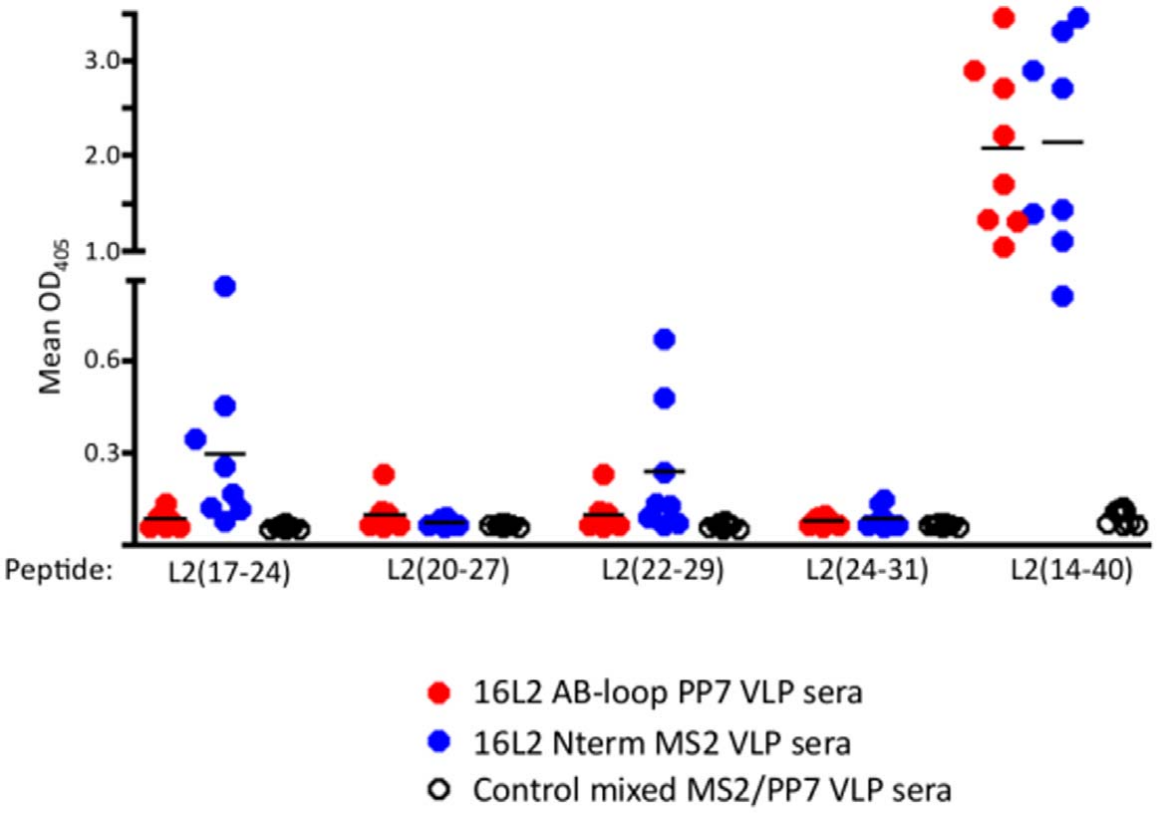
Reactivity of sera with short, linear HPV16 L2 peptides. ELISA plates were coated with 500 ng of streptavidin-conjugated 16L2 peptides (amino acids 17–24, 20–27, 22–29, 24–31, and 14–40) and reacted with a 1∶40 dilution of sera from mice immunized with either 16L2(17–31) Nterm MS2 VLPs or 16L2(17–31) AB-loop PP7 VLPs or MS2/PP7 VLPs. Average ODs (405 nm) from a group of five mice are shown in solid black horizontal lines.

To investigate whether the broader cross-reactivity observed with 16L2(17–31) Nterm MS2 VLP sera is due to its ability to bind to linear or conformational epitopes, 8-mer peptides spanning the 16L2 epitope (amino acids 17–24, 20–27, 22–29, and 24–31) were synthesized and used as target antigens in an ELISA. As shown in Figure 5, neither VLP elicited antibodies that bound strongly to any of the short synthetic peptides. MS2-L2 VLPs elicited antibodies with weak reactivity to L2(17–24) and L2(22–29). These data appear to indicate that both L2-VLPs elicit antibodies that by and large recognize epitopes that are larger than eight amino acids.

**Figure 6:**
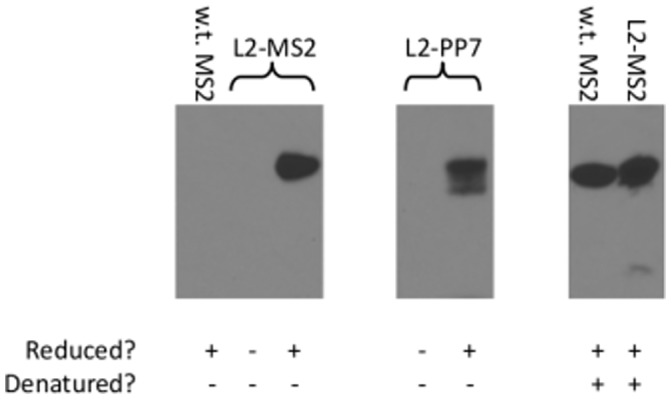
L2 cysteines displayed on PP7 and MS2 VLPs are not reactive with BMCC-biotin. Purified VLPs were mock-treated, DTT-treated and/or denatured prior to incubation with thiol-reactive BMCC-biotin. Proteins were separated by SDS-PAGE, transferred to membranes, and biotinylation was detected using streptavidin-HRP.

The region of L2 that we are targeting contains two cysteine residues (C22 and C28) that are absolutely conserved throughout the *Papillomaviridae* and form a disulfide bond in their native context [24]. To examine the native redox state of the two cysteines within the structural context of bacteriophage VLPs, we performed a thiol-specific biotinylation assay. Purified PP7 and MS2 L2-VLPs were kept in their native state or reduced by DTT prior to chemical biotinylation with a thiol-specific BMCC-biotin reagent. Biotinylated proteins were then detected by streptavidin-HRP staining. Both MS2 and PP7 coat protein contain cysteine residues, but these residues are buried in the particle and were not predicted to be accessible to the BMCC-biotin reagent. As shown in Figure 6, neither native L2-MS2 VLPs nor L2-PP7 VLPs were modified by BMCC-biotin, suggesting that the L2 cysteines were disulfide-bonded. In contrast, control experiments show that denatured wild-type MS2 VLPs, denatured L2-MS2 VLPs, reduced L2-MS2 VLPs, and reduced L2-PP7 VLPs were modified by BMCC-biotin. Thus, these data indicate that cysteines within the L2 peptide form a disulfide bond on both PP7 and MS2 VLPs.

### N-terminal Display of L2 on MS2 Induces Broadly Protective Antibody Responses in an in vivo Pseudovirus Challenge Model

**Figure 7:**
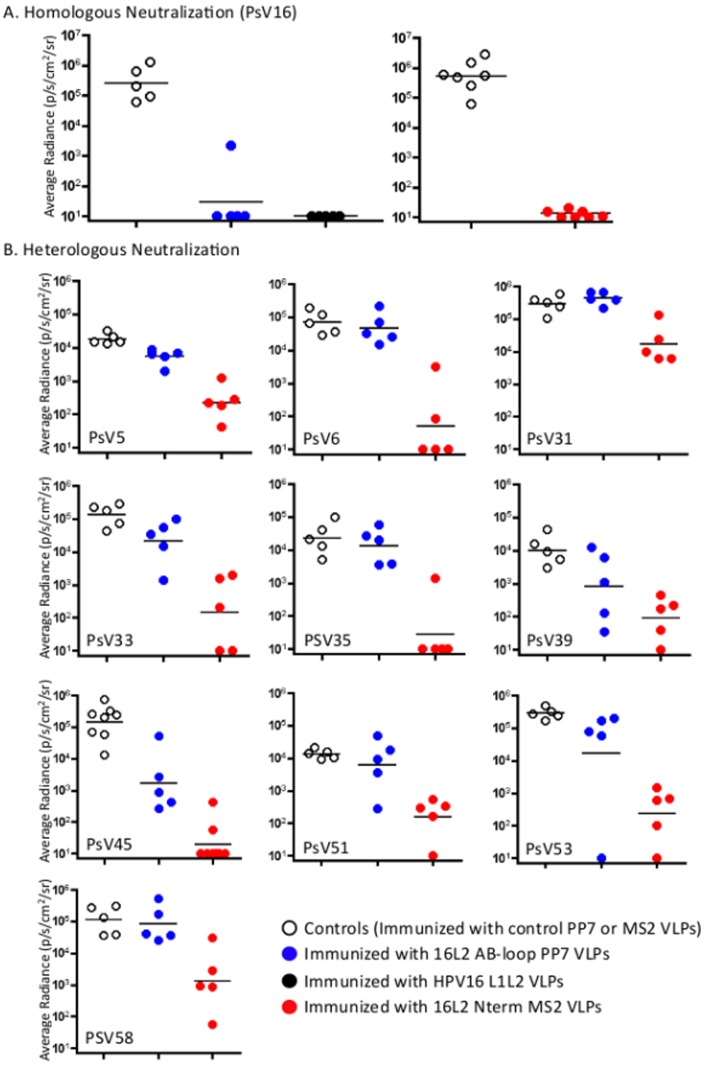
Mice immunized with 16L2(17–31) Nterm MS2 VLPs show broader protection from infection with diverse HPV PsV types compared to those immunized with 16L2(17–31) AB-loop PP7 VLPs. Balb/c mice were immunized (intramuscularly) twice at two weeks intervals with 5 µg of 16L2(17–31) Nterm MS2 VLPs or 16L2(17–31) AB-loop PP7 VLPs or HPV16 L1L2 VLPs or a mixture of MS2/PP7 VLPs. Three to five weeks after the last immunization, mice were challenged with: A) PsV16 and B) PsV5, 6, 31, 33, 35, 39, 45, 51, 53, and PsV58. All challenges were done vaginally except PsV5, which was done intradermally. Forty-eight hours post-challenge, 0.4 mg of luciferin was administered via the same route used for PsV challenge and average radiance (p/s/cm^2^/sr) values for each mouse was determined using Living Image 3.2 software. White-filled black circles represent mice immunized with MS2/PP7 VLPs, black-filled circles represent mice immunized with HPV16 L1L2 VLPs, blue-filled circles represent mice immunized with 16L2(17–31) AB-loop PP7 VLPs, and red-filled circles represent mice immunized with 16L2(17–31) Nterm MS2 VLPs. Black solid lines represent the geometric mean of average radiance.

To determine whether the broader cross-reactivity observed with the insertion of epitope 17–31 at the N-terminus of MS2 coat protein correlated with protection, mice were immunized with VLPs displaying the HPV16 L2 epitope at either the N-terminus of MS2 coat protein [16L2(17–31) Nterm MS2 VLPs] or at the AB-loop of PP7 coat protein [16L2(17–31) AB-loop PP7 VLPs] or control VLPs, and were then challenged with a panel of PsVs (PsV5, 6, 16, 31, 33, 35, 39, 45, 51, 53, and 58) three to five weeks after the 2^nd^ immunization. As expected, mice immunized with either 16L2(17–31) Nterm MS2 VLPs or 16L2(17–31) AB-loop PP7 VLPs showed almost complete protection from high-dose vaginal challenge with homologous HPV16 PsV (Figure 7A). Protection was similar to mice immunized with HPV16 L1L2 VLPs. However, there were dramatic differences in protection from heterologous HPV PsV challenge (Figure 7B and Table 1). Immunization with L2-PP7 VLPs resulted in modest or no protection against heterologous PsV. In contrast, immunization with L2-MS2 VLPs resulted in significant protection from vaginal challenge with nine heterologous types and one intradermal challenge with HPV5 PsV. We observed very strong (80- to 7,190-fold reduction in signal) protection from infection with 9 out of the 10 heterologous PsV types tested. Protection from HPV31 PsV was somewhat weaker (17-fold), but still statistically significant (*p*<0.01, one-tailed *t*-test, see Table 1). Thus, immunization with MS2 VLPs displaying a single HPV16 L2 peptide provided protection against diverse HPV pseudovirus types.

**Table 1:** Summary of *in vivo* HPV PsV challenge experiments.

	16L2(17–31) AB-loop PP7 VLPs		16L2(17–31) Nterm MS2 VLPs	
HPV PsV	*Fold reduction	P-value	*Fold reduction	P-value
16	**6263**	**<0.0001**	**68228**	**<0.0001**
5	**3.4**	**0.002**	**80**	**<0.0001**
6	1.5	0.24	**1430**	**0.0001**
31	0.65	0.14	**17**	**0.0011**
33	6.4	0.028	**910**	**0.0003**
35	1.7	0.25	**840**	**0.0002**
39	12	0.037	**110**	**0.0002**
45	**84**	**0.0003**	**7190**	**<0.0001**
51	2.2	0.19	**88**	**0.0001**
53	16	0.088	**1240**	**<0.0001**
58	1.35	0.35	**86**	**0.002**

* Fold reduction in geometric mean radiance compared to control mice immunized with mixed MS2/PP7 VLPs. Bold values indicate that the reduction in luminescence was statistically significant (*p*<0.01).

## Discussion

The HPV minor capsid protein L2 has great potential as the basis of a second generation HPV vaccine. L2 immunogens can induce antibody responses that are much more broadly neutralizing than the L1-VLPs that make up the current HPV vaccines. However, L2-based vaccines have their own hurdles to overcome in order to be clinically effective. For one, L2 vaccines induce considerably lower neutralizing titers than L1-VLP based vaccines. This raises concerns that the protection provided upon vaccination with L2 will be short-lived. In addition, the sequence of L2 does vary amongst HPV types and, in general, immunization with single L2 peptides typically results in sera that only cross-neutralizes a subset of HPV types. For example, rabbit antisera induced upon immunization with synthetic linear peptides encoding short (15–20 amino acid) peptides spanning the N-terminal domain of HPV16 L2 effectively neutralize HPV16 PsV, but only display varying degrees of cross-neutralizing activity against HPV6, 18, 31, and 58 PsV [15], [25]. The epitope represented by amino acids 17–36 from HPV16 L2 clearly contains the most broadly protective neutralizing epitopes [18]. Nevertheless, even this epitope has its limitations in the amount of cross-protection conferred through vaccination.

We and others have shown that these limitations can be addressed by altering the way that L2 is displayed to the immune system. For example, the immunogenicity of L2 can be improved by the use of potent adjuvants or by increasing the multivalency of L2 through the use of concatemeric constructs [13] or by displaying L2 on the surface of VLPs [12], [20], [26], [27], [28], [29]. In addition, we have shown that immunization of mice with PP7 VLPs displaying L2 results in stable anti-L2 antibody titers that persist for over 15 months after immunization (unpublished data).

The lack of broad cross-reactivity of antibodies induced by individual L2 peptides is exemplified by our previous studies using the PP7 VLP display platform. Display of L2 amino acids 17–31 from individual HPV types on bacteriophage PP7 VLPs induced high-titer type-specific antibodies to HPV peptides but generally low-titer cross-reactive antibodies to heterologous HPV types. These single L2 PP7 VLPs only induced broadly reactive and protective antibody responses when all eight VLPs were mixed together as a single immunogen [12]. Although the mixed L2 PP7 VLPs did not have a negative impact on the immunogenicity of individual L2 VLPs in the mixture (and it is not clear that all eight VLPs were required for protection), a vaccine consisting of a single antigen would be ideal.

In this regard, we explored whether display of L2 at an alternative site on the surface of a bacteriophage VLP could broaden the spectrum of protection provided by a single recombinant L2 VLP. We have identified three different sites on the bacteriophage VLP that allow for surface display of antigens; the N-terminus, the AB-loop, and the C-terminus. We have previously described the insertion of peptides on to the AB-loop of bacteriophage PP7 and MS2 [12], [20], [21]. Although the AB-loop is broadly tolerant of short (6–10 amino acid) peptide insertions, our experience is that larger insertions are more likely to cause assembly defects and prevent the formation of VLPs (unpublished data). Display of peptides at the C-terminus of PP7 and MS2 is possible only by using methods to dramatically reduce the valency of display (unpublished data), which reduces the immunogenicity of the resulting VLPs. Given the success of displaying long peptides at the N-terminus of coat protein of the related RNA bacteriophage AP205 [22], we decided to investigate whether the single-chain dimer of MS2 coat protein would tolerate HPV L2 peptide insertions at its N-terminus and whether display at this site could elicit high-titer antibody responses.

In this study, we have shown that the N-terminus of the MS2 single-chain dimer coat protein can be modified to display HPV16 L2 peptides ranging from 10 to 27 amino acids (but not 52 amino acids) without affecting VLP assembly. These peptides are displayed on the surface of VLPs in a highly immunogenic format; all three L2-MS2 VLPs generated in this study induced high-titer anti-HPV16 L2 IgG antibody responses even at low (sub-microgram) doses without IFA. The different peptides elicited slightly different degrees of cross-reactive antibodies against heterologous HPV L2 peptides; the VLP displaying amino acids 17–31 induced the most broadly reactive antibody response. Strikingly, display at the N-terminus of MS2 elicited much more broadly reactive antibody responses than display of the same peptide in AB-loop of PP7 coat protein. It is likely that this configuration allows more conformational flexibility, perhaps, leading to a better exposure of conserved amino acids in this epitope thereby inducing a more broadly reactive antibody response against a conformational epitope. Correspondingly, mice immunized with 16L2(17–31) Nterm MS2 VLPs were more broadly protected upon vaginal and intradermal challenge with a diverse panel of HPV PsVs.

Thus, in this manuscript we describe a single, potentially low-cost, bacterially-expressed VLP-based vaccine that elicits high-titer broadly neutralizing antibody responses against diverse HPV types at low doses. Bacteriophage VLPs can be expressed at high levels, are stable at room temperature [30], and elicit long-lasting antibody responses. These features may be particularly valuable for use in resource-poor settings, where the vast majority of cases of cervical cancer arise.

## Materials and Methods

### Genetic Insertion of HPV 16L2 Peptides on to the N-terminus of the Single-chain Dimer of MS Coat Protein

L2 sequences representing HPV16 L2 amino acids 20–29, 17–31, 14–40, and 14–65 were cloned onto the amino-terminus of a single-chain dimer version of the MS2 coat protein in plasmid pDSP62 [31] by PCR. Forward PCR primers consisted of nucleotides in the following order: an NcoI restriction site, followed by a start codon and an alanine amino acid, 16L2 peptide sequences, a linker sequence (4–6 amino acids), and a sequence that is complementary to the N-terminus of MS2 juggled codon coat protein (shown diagrammatically in Figure 1). Reverse primer E3.2 (5′ CGGGCTTTGTTAGCAGCCGG 3′), which anneals downstream of a unique BamHI site in the pDSP62 plasmid was used for all PCR amplifications above. PCR fragments were cloned using NcoI/BamHI sites into the pDSP62 expression plasmid. For peptide 14–65 insertion, plasmid pDSP62-16L2(14–40), which contains the nucleotide sequence for L2 peptide 14–40, was used as a template for whole plasmid PCR amplification using phosphorylated primers. All constructs were sequenced to confirm the presence of the L2 peptide inserts.

### Expression, Purification, and Characterization of VLPs

C41 cells (Lucigen) were transformed with recombinant pDSP62-16L2 plasmids and recombinant 16L2-MS2 VLPs were expressed and purified as previously described [12], [20]. Purified recombinant 16L2 Nterm MS2 VLPs were run on a 1% agarose gel stained with ethidium bromide to check for coat protein-encapsidated RNA; the same gel was then stained with coomassie blue to check for the presence of recombinant coat proteins at the same position of encapsidated RNA. Transmission electron microscope (TEM) analyses of recombinant 16L2-MS2 VLPs were conducted as described in Tumban et al., [12] and VLPs were visualized at a magnification of 70,000x. The presence of L2 peptides on the VLPs was determined by Western blotting as follows: 100 ng of each VLP was resolved on a 10% SDS-PAGE in duplicate, transferred to polyvinylidene fluoride membranes (PVDF; Life Sciences) and blocked for two hours with blocking buffer (5% milk in 1X TBS, 0.05% Tween-20). Primary antibodies (polyclonal sera from mice immunized with 16L2 peptide 1–88 or from rabbit immunized with MS2 VLPs) at a 1∶5,000 dilution in blocking buffer were applied to either membrane for 2 hours. Membranes were washed five times and horseradish peroxidase (HRP)-conjugated goat anti-mouse or goat anti-rabbit antibodies at 1∶10,000 dilution in blocking buffer were added to their respective membranes for 1 hour. The membranes were then washed and developed using SuperSignal West Pico Chemiluminescent Substrate (Thermo Scientific) after which they were exposed to Blue Basic Automated Film (GeneMate).

To examine the native redox state of the two cysteines within the structural context of bacteriophage VLPs, L2-VLPs were biotinylated at 1∶50 molar ratio (VLPs:biotin) using 1-Biotinamido-4-[4′-(maleimidomethyl)cyclohexanecarboxamido]butane (BMCC-biotin; Thermo Scientific) for two hours at room temperature. Biotinylated VLPs were then purified from residual BMCC-biotin using the Amicon 100 K MWCO centrifugal filters (Millipore). In some cases, VLPs were reduced or denatured prior to the BMCC-biotin reaction. Reduction was performed by overnight incubation with 10 mM dithiothreitol (DTT) while denaturation was performed by incubating VLPs in 2% SDS for 1 hour at 37°C. Reduced or denatured VLPs were purified by 3X PBS buffer exchanges using Amicon 100 K MWCO centrifugal filters. For western blotting, non-reducing protein loading dye (62.5 mM Tris pH 6.8, 0.05% bromophenol blue, 10% glycerol, 2% SDS) was added to 500 ng of each VLP, heated for 5 minutes and resolved under denaturing conditions on a 12% SDS PAGE gel. VLPs were then transferred to a PVDF membrane and blocked as described above. Thio-biotinylated VLPs were probed with 1∶5,000 dilution of streptavidin-conjugated HRP for two hours and the membrane was developed as described above.

### Immunizations and Characterization of Antibody Responses

All animal work was done in accordance with the National Institutes of Health and the University of New Mexico Institutional Animal Care and Use Committee (UNM IACUC) guidelines and was approved by the UNM IACUC (protocol 12-100827-HSC). Groups of Balb/c mice were immunized twice intramuscularly (i.m.) at a two-week interval with 5 µg of 16L2 Nterm MS2 VLPs (displaying L2 amino acids 20–29, 17–31, or 14–40), 16L2 AB-loop PP7 VLPs (displaying L2 amino acids 17–31), or, as negative controls, a mixture of wild-type MS2 and PP7 VLPs. Vaccine was prepared with incomplete Freund’s adjuvant (IFA). Another group of Balb/c mice was immunized i.m. with 250 ng of 16L2(17–31) Nterm MS2 VLPs without IFA and were boosted 4 weeks later with 500 ng without IFA. Two weeks after the second immunization, sera were collected (except for the mice immunized without IFA whereby sera was collected at one and three weeks after first immunization and then one week after second immunization) and anti-L2 IgG titers were determined by peptide-based ELISA using disulfide-constrained L2 peptides representing amino acids 14–40 from HPV1, 5, 6, 16, and 18 (as described [12]) or biotinylated 8-mer peptides that span epitope 17–31 of HPV 16L2 (American Peptide Company). The biotinylated peptides were used as target antigens for ELISA as follows. Peptides were mixed with streptavidin at a molar ratio of 8∶1 and incubated at room temperature for 30 minutes. 500 ng of each streptavidin-conjugated L2 peptide was coated on ELISA plates and the rest of the ELISA procedure was conducted as previously described [12].

### HPV Pseudovirus Production and Purification

HPV5, 6, 16, 31, 33, 35, 39, 45, 51, 53, and 58 PsVs with encapsidated reporter plasmid (pClucf) encoding both luciferase and green fluorescence protein (GFP) genes were produced in 293TT cells as previously described [12], [32], [33]. PsV-infectivity titer was characterized using flow cytometry by determining the fraction of 293TT cells expressing the GFP protein. HPV5, 6, 16, 31, 45, and 58 L1/L2 expression vectors and reporter plasmids (http://home.ccr.cancer.gov/lco/packaging.htm) were generously provided by Chris Buck, Susana Pang, John Schiller, Martin Muller, and Tadahito Kanda. pVitro plasmids for HPV33, 35, 39, 51, and 53 L1/L2 expression were kindly provided by Richard Roden.

### HPV Pseudovirus Challenge

Prior to challenge, groups of five female Balb/c mice were given two i.m. immunizations with 5 µg of 16L2(17–31) Nterm MS2 VLPs, 16L2(17–31) AB-loop PP7 VLPs, HPV16 L1L2 VLPs, or with equal mixture of wild-type MS2/PP7 VLPs with IFA. Three to five weeks after the last immunization, mice were treated with 3 mg of Depo-Provera (Pharmacia Corp) except the group for intradermal challenge. Five days post-Depo-Provera treatment, mice were challenged either cervicovaginally or intradermally with HPV PsVs as previously published [12], [34], [35], [36] using 6.0×10^4^–3.0×10^6^ PsV infectious units. Forty-eight hours post-PsV challenge, mice were vaginally instilled or injected intradermally with 0.4 mg of luciferin (Caliper Life Sciences) and three minutes later their images were taken (with a five minute exposure) using a Caliper IVIS Lumina II (Caliper Life Sciences). Average radiance (p/s/cm2/sr) was determined from the images by drawing equally sized regions of interests surrounding the site of PsV instillation. Statistical significance was determined by one-tailed unpaired t-test.
